# Synthesis, Characterization and Photophysical Properties of Pyridine-Carbazole Acrylonitrile Derivatives

**DOI:** 10.3390/ma4030562

**Published:** 2011-03-11

**Authors:** Enrique Pérez-Gutiérrez, M. Judith Percino, Víctor M. Chapela, Margarita Cerón, José Luis Maldonado, Gabriel Ramos-Ortiz

**Affiliations:** 1 Centro de Química, Instituto de Ciencias, Universidad Autónoma de Puebla, Complejo de Ciencias, ICUAP, Edif. 103F, 22 Sur y San Claudio, C.P. Puebla 74570, Puebla, Mexico; E-Mails: cuper_enrique@msn.com (E.P.-G.); vchapela@siu.buap.mx (V.M.C.); mceron@siu.buap.mx (M.C.); 2 Centro de Investigaciones en Óptica A.C. (CIO), Lomas del Bosque # 115, Col. Lomas del Campestre, C.P. 37150, León, Guanajuato, Mexico; E-Mails: jlmr@cio.mx (J.L.M.); garamoso@cio.mx (G.R.-O.)

**Keywords:** conjugated compounds, ciano derivatives, optical properties, fluorescent compounds, powder fluorescent quantum yield

## Abstract

We synthesized three novel highly fluorescent compounds, 2-(2’-pyridyl)-3-(N-ethyl-(3’-carbazolyl))acrylonitrile, 2-(3”-pyridyl)-3-(N-ethyl-(3’-carbazolyl))acrylonitrile, and 2-(4-pyridyl)-3-(N-ethyl-(3’-carbazolyl))acrylonitrile by Knoevenagel condensation. The first two were synthesized without solvent in the presence of piperidine as a catalyst; the third was synthesized without a catalyst and with N,N-dimethylformamide as a solvent. In solution, the molar absorption coefficients showed absorptions at 380, 378, and 396 nm, respectively; in solid state, absorptions were at 398, 390, and 442 nm, respectively. The fluorescence emission was at 540, 540 and 604 nm, respectively, the 2-(4-pyridyl)-3-(N-ethyl-(3’-carbazolyl))acrylonitrile showed a red shift in the emission of 64 nm compared to the other two compounds. The fluorescence quantum yield for the compounds in powder form showed values of 0.05, 0.14, and 0.006, respectively; compared with the value measured for the Alq_3_ reference, 2-(3”-pyridyl)-3-(N-ethyl-(3’-carbazolyl))acrylonitrile had a lightly higher value. The third harmonic generation measurement for 2-(2’-pyridyl)-3-(N-ethyl-(3’-carbazolyl))acrylonitrile yielded a χ^(3)^ value of 5.5 × 10^−12^ esu, similar to that reported for commercial polymers.

## 1. Introduction

Low molecular weight and oligomeric, organic compounds with optical or electrical properties have been widely used as dyes in organic electronic devices, including light emitting diodes (OLEDs), solar cells, organic semiconductor lasers, *etc*. [1,2,3,4,5,6]. Typically, the dyes have a donor-acceptor structure that controls their photophysical properties [2,7,8]. Carbazole is an organic compound that is well known for its electron-donor properties [9]; OLEDs have used carbazole based dimers, oligomers, and polymers as blue, white, green, and red emitters [10,11,12,13,14]. Carbazole polymers are also used in the hole transport layer [15,16] of organic field effect transistors [17,18] and photovoltaic cells [19,20]. On the other hand, poly(phenylenevinylene) and carbazole compounds (low molecular weight and polymers) are of great interest because they belong to the family of organic materials that possess strong π-electron conjugation; *i.e.*, they extend electron delocalization. Recent reports have shown that several of these derivatives have third-order nonlinear optical (NLO) properties [21,22,23,24,25,26,27]. It is thought that strong π-electron conjugation is crucial for attaining high optical nonlinearities; thus, it is of great interest to identify or understand the structure-property relationships of these derivatives for photonic applications [28,29,30]. This knowledge would facilitate the rational design of new second- and third-order NLO materials based on both low molecular weight molecules and polymers. Adès *et al*. described a donor-acceptor dye based on a carbazole unit linked through a vinylene conjugation to electron-acceptor groups, like nitrile (-CN), ethylester (-COOEt), phenyl (-Ph), or *para*-nitrophenyl (-PhNO_2_) [8]. They reported that these compounds underwent a red shift in absorption and emission that was associated with the electron-withdrawing character of the groups. For the -Ph group, the absorption showed a maximum at 385 nm; for the -CN, -COOEt, and -PhNO_2_ groups, the maxima were at 397, 416, and 428 nm, respectively. The λ_emission_ was at 490 nm for both the -COOEt and -Ph groups, and at 530 and 585 for the -CN and -PhNO_2_ groups, respectively.

On the other hand, the pyridine is an important electron-acceptor group, due to its high electron affinity. Dailey *et al.* described the poly(2,5-pyridinediyl) (PPY) as an efficient electron transport layer in bilayer polymeric LEDs. The OLEDs with a PPY layer exhibited an external quantum efficiency 60-times greater than that of similar devices without a PPY layer [31]. Epstein *et al*. described poly(*p*-pyridine)- and poly(*p*-pyridylvinylene)-based polymers as emissive layers in light emitting devices [32].

Su, *et al*. [33] showed that the combination of a carbazole electron-donor and a pyridine electron-acceptor could give bipolar host materials; for example, 2,6-bis[(3-(carbazol-9-yl)phenyl)]pyridine and 3,5-bis[(3-(carbazol-9-yl)phenyl)]pyridine. These host materials were doped with iridium(III) bis[(4,6-(difluorophenyl)pyridinato-*N*,C^2’^)]picolinate to create phosphorescent OLEDs.

The route of synthesis is very important for obtaining the desired dye structures for a specific application. Some dyes with donor-acceptor structures have been formed in a Heck coupling reaction with palladium-catalysis [2], others were formed in the Knoevenagel condensation [8]. Our group previously synthesized different conjugated compounds with the Knoevenagel condensation, without a catalyst, and under solvent-free conditions [34,35,36,37]. This method was successfully used to synthesize α,β-diphenylacrylonitrile; α-(2-pyridyl)-β-(phenyl)acrylonitrile; α-(3-pyridyl)-β-(phenyl)acrylonitrile; α-(phenyl-β-(2-pyridyl)acrylonitrile; 1,4-bis[1-(2-cyano-2-phenyl)vinyl]benzene; 1,4-bis[1-(2-cyano-2’-pyridyl)vinyl]benzene; and 1,4-bis[1-(2-cyano-3-pyridyl)vinyl]benzene, among other compounds [37].

In the present work, we used the Knoevenagel condensation for the synthesis of three novel acrylonitrile derivatives from N-ethyl-3-carbazolecarboxaldehyde and pyridineacetonitrile to form a donor-acceptor structure. The synthesized compounds were: (**I**) 2-(2’-pyridyl)-3-(N-ethyl-(3’-carbazolyl))acrylonitrile, (**II**) 2-(3’-pyridyl)-3-(N-ethyl-(3”-carbazolyl))acrylonitrile, and (**III**) 2-(4-pyridyl)-3-(N-ethyl-(3’-carbazolyl))acrylonitrile (Scheme I). The aim of the synthesis was to obtain fluorescent compounds that exhibit a strong emission and bathochromic effect which was correlated to their optical properties with the structure of the compounds, especially the position of the N in the pyridine ring and the CN group attached to the double bond.

**Scheme I:**
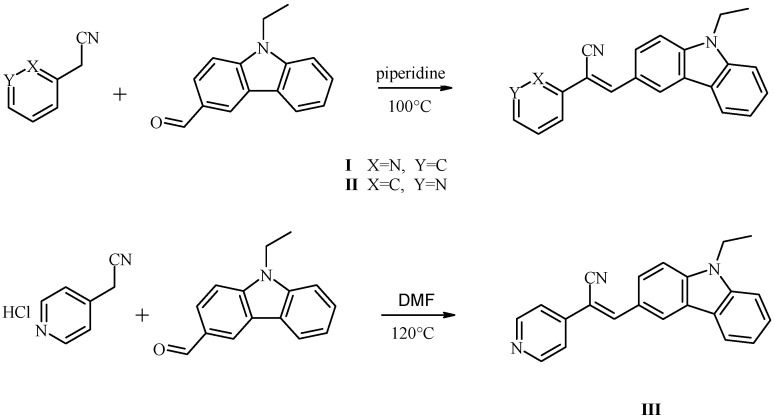
Synthetic routes of 2-(2’-pyridyl)-3-(*N*-ethyl-(3’-carbazolyl))acrylonitrile (**I**), 2-(3’-pyridyl)-3-(*N*-ethyl-(3”-carbazolyl))acrylonitrile (**II**), and 2-(4-pyridyl)-3-(*N*-ethyl-(3’-carbazolyl)) acrylonitrile (**III**).

## 2. Results and Discussion

### 2.1. FT-IR and NMR Characteristics

FT-IR characterization of compounds **I**–**III** showed similar characteristic bands in the region of 1628–1629 cm^−1^. These were assigned to the ν(C=C) vibrations of the alkene double bond, which is conjugated with the aromatic ring, and the CN attached to the double bond [38]. The bands at 745 cm^−1^, 747 cm^−1^, and 744 cm^−1^ for compounds **I**–**III**, respectively, were assigned to the δ(C-H) vibrations of the double bond. The bands at 2210, 2210, and 2213 cm^−1^, respectively, were due to the C≡N stretching vibrations. The ^1^H NMR data showed that the principal evidence [38] for the structures of the prepared compounds were the singlets at δ: 8.652, 8.612, and 8.833 ppm for **I**, **II**, and **III**, respectively, due to the proton attached to the double bond. The ^13^C NMR spectra exhibited signals at δ 119.2 and 118.6 for **I** and **II**, respectively; these were assigned to the C atom of the nitrile group. These values are in good accordance with those recently reported for some nitriles [39,40,41].

### 2.2. Absorption

**Figure 1:**
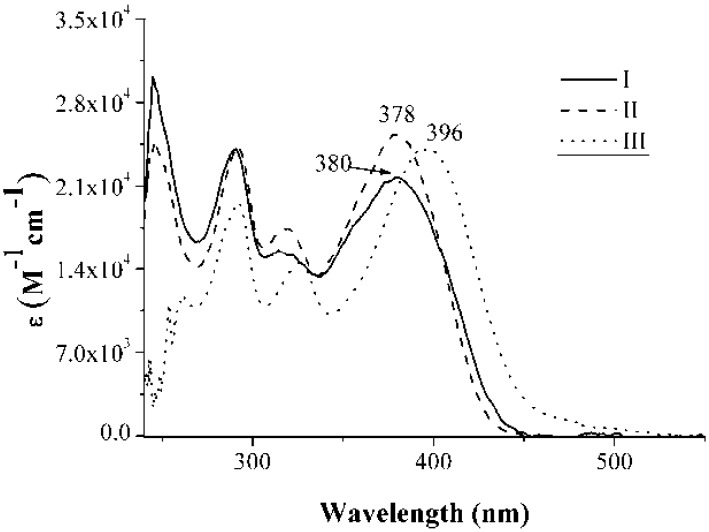
Molar absorptivity of compounds **I**, **II**, and **III**.

**Figure 2:**
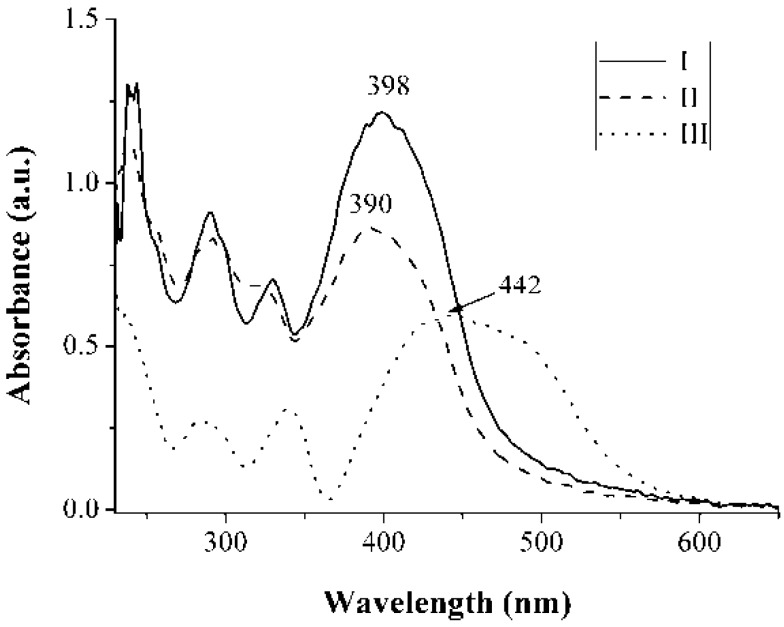
Absorbance in the solid state for compounds **I**, **II**, and **III**.

The molar absorptivity (ε) spectra of compounds **I** and **II** in chloroform and of compound **III** in DMSO are given in Figure 1. All three compounds displayed strong absorption (ε > 20,000); the highest was exhibited by compound **II**. The bands observed below 350 nm are characteristic of the pure carbazole unit; the broad absorption, with λ_max_ values of 380, 378, and 396 nm for compounds **I**, **II**, and **III**, respectively, was attributed to π-π* transitions. A bathochromic shift was observed for the -*para* position of the pyridine ring. Figure 2 shows the UV-Vis absorption in solid state with KBr as host material. In the solid state, the compounds exhibited a bathochromic shift of around 18–20 nm for **I** and **II**, compared to their absorptions in solution (Figure 1). For **III**, the band at 396 nm presented a red shift of around 50 nm and a widening of the band in the solid state compared to the absorption in solution (Figure 2). The broadened band and the pronounced red shift absorption could be explained by the formation of J-aggregates, which form by self-association of dyes in the solid state due to strong intermolecular Van der Waals-like attractive forces between the molecules [42,43]. The J-aggregates are known to cause a bathochromic shift in the absorption spectrum.

### 2.3. Fluorescence

**Figure 3:**
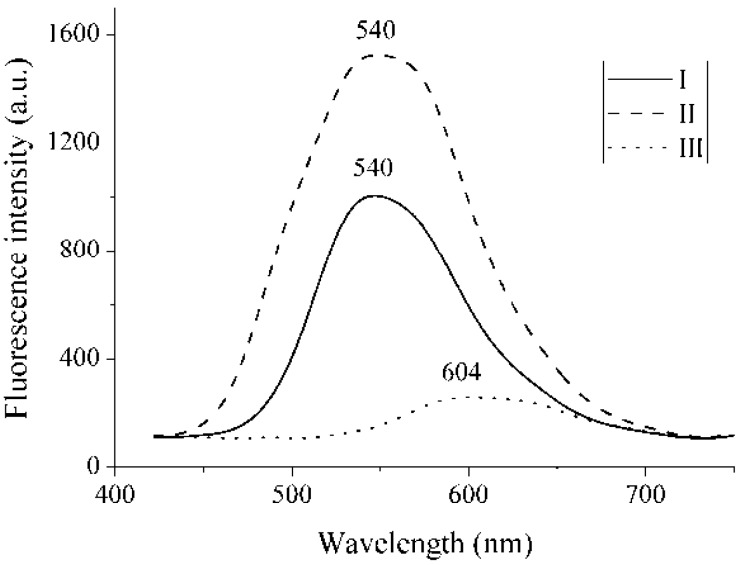
Fluorescence signals for compounds **I**, **II**, and **III** in the solid state.

Figure 3 shows the fluorescence signals for the studied compounds in the solid state. All three compounds showed very weak fluorescence in solution; however, in the solid state, they displayed strong fluorescence λ_f_ at 540 nm for compound **I** and **II** and at 604 for compound **III**. The signal for compound **II** was higher than those for compound **I** or **III**. For compounds **I** and **II**, the strong emission could be a consequence of the phenomena known as aggregation-induced emission (AIE) [44,45,46]. AIE could be caused by restricted intramolecular rotations that form non-planar structures, which reduce intermolecular interactions and excimer formation; as a result, the fluorescence efficiency is increased. On the other hand, the effects of intramolecular planarization could cause the formation of J-aggregates in the solid state, which would induce a red shift and enhance the solid state emission. For compound **III**, the behavior of compound **III**, large red shift of emission and significant low intensity of fluorescence could have two possible explanations; first, the observed results may be attributed to aggregate formation and exciton coupling (excimer). Alternatively, the red-shift in the absorption and emission spectra may be attributed to planarization of the molecule. The formation of excimers is known to drastically decrease the fluorescence quantum yield in the solid state due to the greater number of non-radiative decay pathways during the depopulation of the excited state. Also the red shift of the emission for compound **III**, compared with **I** and **II**, could be due to its donor-acceptor structure associated with the *-para* position of the pyridine ring.

Many groups have attempted to measure the absolute Ф_f_ of compounds in the solid state, particularly for thin solid films [47,48,49,50,51]. However, it is well known that this measurement is complicated due to the difficulties in determining the angular distribution of the emission, reflectivity, and absorbance in solid state compounds, like films of polymeric semiconductors. In the present work, we determined the Ф_f_ for the three compounds in powder form. For the determination of Ф_f_, the experimental setup was similar to that described by De Mello, except that compounds **I**–**III** were not prepared in films; instead, the powdered compounds were placed into Pyrex tubes. First, the laser emission intensity was measured for the empty tube, located inside the integrating sphere. Then, the compound powder was placed inside the tube and the fluorescence spectra were measured as described by De Mello. The Ф_f_ value of Alq_3_, taken as a reference, has been reported for films to be approximately 0.20–0.25 [49,50]. For our experimental setup, the Alq3 Ф_f_ value was 0.11, which we used for the reference. The values measured for compounds **I**, **II**, and **III** were 0.05, 0.14, and 0.006, respectively. Compound **II** had the highest value, slightly higher than that of the Alq_3_ reference.

**Figure 4:**
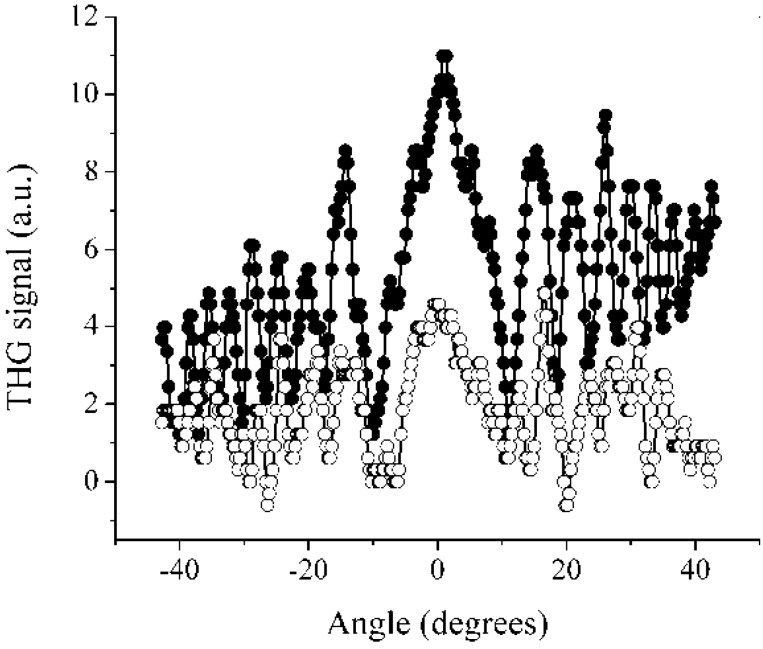
Third harmonic generation (THG) Maker-fringe pattern for a 92 nm-thin polystyrene polymer film of **I** doped with 30 wt. % of compound (filled circles) and for a 1 mm-thick substrate without a film deposited on it (open circles). The fundamental wavelength was 1200 nm.

Next, we measured the third harmonic generation (THG) property for compound **I**. Figure 4 shows the THG Maker-Fringe pattern for a compound **I** film doped with PS. As reference, Figure 4 also includes the THG pattern measured from the fused silica substrate alone (thickness: 1 mm). These data were obtained at the fundamental near IR wavelength of 1200 nm (THG signal at 400 nm). From these data, we estimated that the third-order nonlinear susceptibility (*χ*^(3)^) of the films was in the order of 10^−12^ esu at this fundamental wavelength. The absorption coefficient was taken into account according to Equation 1. For the well known PPV polymer, *χ*^(3)^ was reported to be in the order of 10^−10^ to 10^−11^ esu [24]. For some oligomers of PPV (PV-n), *χ*^(3)^ values were reported in the order of 10^−11^ to10^−13^ esu [25,26]. Therefore, the *χ*^(3)^ value for compound **I** of 5.5 × 10^−12^ esu, estimated from the THG Maker-Fringe technique, was consistent with those reported in the literature.

In the Maker-fringe technique, the third-harmonic peak intensity from the substrate-film structure, *I_f_^3ω^*, is compared to that produced from the substrate alone, *I_s_^3ω^*. Then, the non-linear susceptibility, *χ*^(3)^, in a film of thickness, *L_f_,* is determined as follows:

(1) $$\chi^{\left(3\right)}=\chi_{s}^{\left(3\right)}\frac{2}{\pi }L_{c,s}(\frac{\alpha /2}{1-\text{exp}(\alpha L_{f}/2)}){\left(\frac{I_{f}^{3\omega }}{I_{s}^{3\omega }}\right)}^{1/2}$$

where *χ_s_*^(3)^ and *L_c,s_* are the non-linear susceptibility and coherence length, respectively, for the substrate at the fundamental wavelength, and α is the film absorption coefficient at the harmonic wavelength [52]. In our calculation, we used *χ_s_*^(3)^ = 3.1 × 10^−14^ esu and *L_c,s_* = 9.0 µm for the fused silica substrate [52]. Our samples satisfied the condition that *L_f_* << *L_c,s_*, in which Equation 1 is valid.

## 3. Experimental Section

### 3.1. Materials and Measurements

2-pyridylacetonitrile, 3-pyridylacetonitrile, 4-pyridylacetonitrile hydrochloride, and piperidine were procured from Aldrich Chemical Co.; N-ethyl-3-carbazolecarboxaldehyde was acquired from Acros Organics; all chemicals were used as received.

The IR spectra of the products were recorded on a Vertex (model 70, Bruker Optics, Germany) 750 FT-IR spectrophotometer by attenuated total reflectance (ATR). ^1^H NMR and ^13^C NMR spectra were obtained in CDCl_3_ and DMSO-*d_6_* on a Varian 400 MHz NMR spectrometer (Varian NMR, Walnut Creek, CA, USA). The electron ionization (EI) spectra were acquired on a Jeol MStation 700-D mass spectrometer (Jeol USA, Peabody, MA, USA). The UV-Vis and fluorescence were acquired with a Spectrometer SD2000 (Ocean Optics, Dunedin, FL, USA). For compounds in solution, absorption was measured at room temperature in CHCl_3_ and DMSO. For compounds in solid state, absorption was measured on pellets prepared with KBr. A UV/Vis DT 1000 CE light source (Analytical Instrument Systems, Inc., Flemington, NJ, USA) was used for measuring absorption. The molar absorption coefficient (ε) was calculated according to the Beer-Lamber law A = εcl [53]. The excitation source for measuring the fluorescence and fluorescence quantum yield (Φ_f_) was a laser diode at a wavelength of 405 nm. The Φ_f_ was measured on compounds in powder form with the experimental setup described by De Mello *et al*. [47], these were compared with the quantum yield of Alq_3_ as reference. The non-linear optical measurements were performed in solid state (solid films) with the guest (compound)-host (polymer) approach. Briefly, polystyrene (PS) and the test compound were mixed at a 70:30 wt% ratio, respectively, in chloroform. The solid films were deposited on fused silica substrates (1 mm-thick) with the spin coating technique. The prepared films had typical thicknesses, between 90 and 120 nm, with good optical quality. The third harmonic generation (THG) Maker-fringes procedure was carried out according to previously reported methods [52,54]. The third-harmonic beam that emerged from the films as a bulk effect was separated from the pump beam with a color filter and was detected with a photomultiplier tube and a Lock-in amplifier. The THG measurements were performed for incident angles in the range from −40° to 40° with steps of 0.27 degrees. All the experiments were computer-controlled.

### 3.2. Synthesis and Characterization of Novel Acrylonitrile Compounds

We synthesized compounds (**I**) 2-(2’-pyridyl)-3-(N-ethyl-(3’-carbazolyl))acrylonitrile, (**II**) 2-(3’-pyridyl)-3-(N-ethyl-(3”-carbazolyl))acrylonitrile, and (**III**) 2-(4-pyridyl)-3-(N-ethyl-(3’-carbazolyl)) acrylonitrile with the Knoevenagel condensation as shown in Scheme I.

The condensation reaction conditions were as follows: (**I**) 0.75 ml (6.8 mmol) of 2-pyridylacetonitrile, 1 g (4.47 mmol) of N-ethyl-3-cabazolecarboxaldehyde, and 0.5 ml (5.06 mmol) of piperidine were combined in a flask; (**II**) 0.35 ml (3.25 mmol) of 3-pyridylacetonitrile, 0.5 g (2.23 mmol) of N-ethyl-3-cabazolecarboxaldehyde, and 0.25 ml (2.53 mmol) of piperidine were combined in a flask. The piperidine acted as catalyst and solvent. The reactions were refluxed with stirring for 24 h at 100 °C. During the reaction, the mixtures appeared oily, and the color changed from colorless, to yellow, to brown. The reactions were distilled after the reaction time in order to eliminate the piperidine, until the precipitate formed. The precipitates were purified by recrystallization with cyclohexane. The final products were (**I**) a yellow powder, yield 29%, m.p. 110–111 °C; and (**II**) a yellow powder, yield 25.7%, m.p. 115–117 °C. The products were soluble in moderately polar solvents. The spectroscopic properties were as follows (with carbon locations as shown in Scheme II):

**Scheme II:**
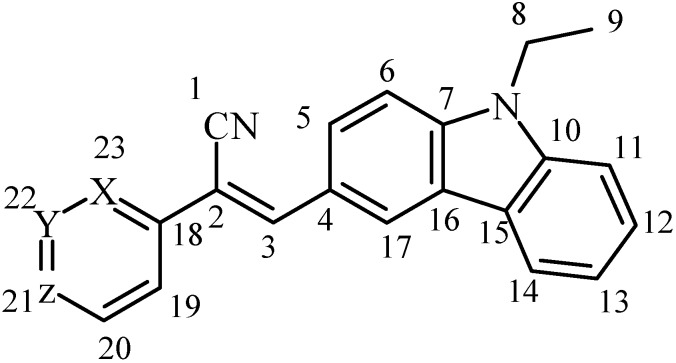
Identification of carbons for ^13^C NMR.

**I**: ^1^H NMR (400 MHz, CDCl_3_, *δ*, ppm): 8.749–8.740 (d, 1H, H_22_), 8.666-8.637 (m, 1H, H_21_), 8.652 (s, 1H, H_3_), 8.241-8.209 (dd, 1H, H_5_), 8.166–8.138 (dd, 1H, H_6_), 7.772 (s, 1H, H_17_), 7.776–7.758 (m, 1H, H_19_), 7.512–7.230 (m, 5H, H_20_, H_11–14_), 4.366–4.334 (m, 2H, H_8_), 1.459–1.435 (t, 3H, H_9_). ^13^C NMR (400 MHz, CDCl_3_, *δ*, ppm):119.2 (C_1_), 105.5 (C_2_), 146.7 (C_3_), 124.6 (C_4_),121.0 (C_5_), 109.2 (C_6_), 141.8 (C_7_), 38.0 (C_8_), 14.1 (C_9_), 140.8 (C_10_), 109.1 (C_11_), 120.2 (C_12_), 122.2 (C_13_), 126.7 (C_14_), 123.1 (C_15_), 123.6 (C_16_), 128.2 (C_17_), 152.1 (C_18_), 123.9 (C_19_), 137.7 (C_20_), 122.9 (C_21_), 149.5 (C_22_). IR (KBr, cm^−1^): 3051 (w), 2979 (m), 2933 (w), (CH, Py, Cz), 2210 (s) (C≡N), 1628 (w, C=C), 1585 (s), 1496 (m), 1468 (s), 1435 (m), (C=C, C=N, Py, Cz), 1233 (s, C-N, Cz), 745 (s, δ(C-H)). EI, *m*/*z* (%, I_r_): 323 (57) (M^+^), 322 (52), 308 (17), 293 (7), 281 (4), 153, (5), 28 (8), 8 (30).

**II**: ^1^H NMR (300 MHz, CDCl_3_, *δ*, ppm): 8.944 (s, 1H, H_23_), 8.612 (s, 1H, H_3_), 8.596–8.584 (m, 1H, H_21_), 8.145-8.096 (m, 2H, H_19_, H_5_), 7.960–7.940 (dd, 1H, H_6_), 7.674 (s, 1H, H_17_), 7.512–7.292 (m, 5H, H20, H_11-14_), 4.346–4.328 (m, 2H, H_8_), 1.455–1.419 (t, 3H, H_9_). ^13^C NMR (400 MHz, CDCl_3_, *δ*, ppm):118.6 (C_1_), 104.0 (C_2_), 145.1 (C_3_), 124.4 (C_4_),121.0 (C_5_), 109.2 (C_6_), 141.6 (C_7_), 38.0 (C_8_), 14.1 (C_9_), 140.7 (C_10_), 109.1 (C_11_), 120.2 (C_12_), 122.2 (C_13_), 126.8 (C_14_), 123.1 (C_15_), 123.5 (C_16_), 127.5 (C_17_), 131.5 (C_18_), 123.8 (C_19_), 133.3 (C_20_), 123.0 (C_21_), 149.4 (C_22_). IR (KBr, cm^−1^): 3052 (m), 2968 (m), 2929 (w), (CH, Py, Cz), 2210 (s) (C≡N), 1629 (w, C=C), 1589 (s), 1495 (s), 1473(m), (C=C, C=N, Py, Cz), 1235 (s, C-N, Cz), 747 (s, δ(C—H)). EI, *m*/*z* (%, I_r_): 323 (98) (M^+^), 322 (14), 309 (17), 308 (67), 293 (9), 266 (5), 153 (7), 18 (17).

To obtain compound **III**, 0.3462 mg (2.23 mmol) of 4-pyridylacetonitrile hydrochloride and 0.5 g (2.23 mmol) of N-ethyl-3-cabazolecarboxaldehyde were dissolved in 10 mL of dimethylformamide (DMF). The reaction was refluxed with stirring for 40 h at 120 °C. During the reaction, the mixture appeared oily, and the color changed from red-pink to brown. The reaction was distilled after the reaction time in order to eliminate the DMF, until the precipitate formed. The precipitate was treated with MeOH to eliminate the DMF hydrochloride formed; the insoluble solid was purified by recrystallization with a mixture of H_2_O:C_3_H_6_O (1:1) to give an orange-red crystal, yield 22.5%, m.p. 215–217 °C. Compound **III** was soluble in: δEtOH, δMeOH, δDMF and δDMSO and completely soluble in excess ΔDMF, ΔDMSO. The spectroscopic properties were as follows:

**III**: ^1^H NMR (300 MHz, CDCl_3_, *δ*, ppm): 8.833 (s, 1H, H_3_), 8.765–8.751 (d, 2H, H_20_, H_22_), 8.590 (s, 1H, H_17_), 8.285–8.258 (dd, 1H, H_11_), 8.145–8.125 (d, 1H, H_5_), 7.943–7.928 (d, 2H, H_19_-H_23_), 7.846–7.824 (d, 1H, H_14_), 7.713–7.693 (d, 1H, H_6_), 7.560–7.539 (m, 1H, H_12_), 7.326–7.307 (m, 1H, H_13_) 4.530–4.494 (m, 2H, H_8_), 1.356–1.320 (t, 3H, H_9_). IR (KBr, cm^−1^): 3038 (w), 2973 (m), 2929 (w), (CH, Py, Cz), 2213 (s) (C≡N), 1624 (w, C=C), 1568 (s), 1499 (m), 1443 (m), (C=C, C=N, Py, Cz), 1231 (s, C-N, Cz), 744 (s, δ(C-H)). EI, *m*/*z* (%, I_r_): 323 (90) (M^+^), 309 (38), 308 (90), 270 (17), 252 (18), 235 (14), 208 (13), 129 (14), 44 (23), 38 (39), 36 (100), 35 (18).

## 4. Conclusions

We synthesized three novel fluorescent pyridine-carbazole based compounds with –CN attached to C=C on the acetonitriles at the *-o*, *-m*, or *-p* position in the pyridine ring. The synthesis of the compounds was carried out with piperidine or DMF as catalyst and solvent. The compounds were characterized with spectroscopic techniques. Optical characterization showed an effect due to the position of the bound moiety in the pyridine ring. The emission could only be observed and measured when the compounds were in the solid state; this could be attributed to the phenomenon of aggregation-induced emission. The quantum yield efficiency was measured in the solid state because, in solution, the compounds did not exhibit a distinguished fluorescence signal; the highest value was for the*—meta* position. Finally, THG measurements for compound **I** showed an interestingly high value for *χ*^(3)^, which was similar to that reported for commercial polymers; even thought the compound **I**, is of low molecular weight.
